# Placental Vitamin D-Binding Protein Expression in Human Idiopathic Fetal Growth Restriction

**DOI:** 10.1155/2017/5120267

**Published:** 2017-02-15

**Authors:** Alice F. Wookey, Tejasvy Chollangi, Hannah E. J. Yong, Bill Kalionis, Shaun P. Brennecke, Padma Murthi, Harry M. Georgiou

**Affiliations:** ^1^Department of Maternal-Fetal Medicine, Pregnancy Research Centre, Royal Women's Hospital, Parkville, VIC 3052, Australia; ^2^Department of Obstetrics and Gynaecology, University of Melbourne, Royal Women's Hospital, Parkville, VIC 3052, Australia; ^3^Department of Medicine, School of Clinical Sciences, Monash University, Clayton, VIC 3168, Australia

## Abstract

Vitamin D-binding protein is a multifunctional serum protein with multiple actions related to normal health. Vitamin D-binding protein transports vitamin D and influences the metabolism of this key hormone but it also has additional immunomodulatory and actin-clearing properties. We investigated whether vitamin D-binding protein expression is altered in fetal growth restriction-associated placental dysfunction. Protein was extracted from 35 placentae derived from 17 healthy control subjects and 18 gestation-matched subjects with fetal growth restriction (FGR). FGR subjects were further subdivided as idiopathic (*n* = 9) and nonidiopathic (*n* = 9). Vitamin D-binding protein and 25(OH) vitamin D were measured by ELISA and normalized to protein concentration. The results showed significantly reduced levels of placental vitamin D-binding protein (control versus FGR, *p* < 0.05, Student's *t*-test) that were strongly associated with idiopathic fetal growth restriction (*p* < 0.01, Kruskal-Wallis), whereas levels of vitamin D-binding protein were not associated with placental 25(OH) vitamin D stores (*p* = 0.295, Pearson's correlation). As such, vitamin D-binding protein may be a factor in unexplained placental dysfunction associated with idiopathic fetal growth restriction and may potentially serve as a biomarker of this disease.

## 1. Introduction

Fetal growth restriction (FGR) is a significant public health problem, particularly because of its association with perinatal mortality and long-term metabolic disease. Despite a prevalence of approximately 5% of all pregnancies, there are no treatments that alter the course of FGR other than delivery, which is often preterm [1]. Thus, obstetricians are forced to balance the risks of iatrogenic prematurity against further developmental damage in utero.

Whilst well established risk factors (e.g., maternal hypertension, toxin exposure, and fetal genetic abnormalities) are identifiable in some cases of FGR, most arise from unexplained placental dysfunction caused by unknown insults during placental development and are categorized as “idiopathic” [1].

Many observational studies link FGR with low levels of vitamin D [2], which is a critical hormone in placental development and function. In particular, vitamin D acts as a key regulator of implantation, inflammation, and production of important pregnancy hormones [3]. Recent data suggest that vitamin D activity is affected by binding to its major carrier, vitamin D-binding protein (VDBP). Vitamin D metabolite levels are influenced by VDBP concentration and genotype in serum [4], and there is evidence that this holds at a tissue level in the placenta [5]. In addition, VDBP regulates global placental function and acts as a determinant of fetal nutrient delivery and long-term metabolic programming [6].

Vitamin D-binding protein (VDBP) is a 58 kDa protein of the albumin superfamily, mainly produced by hepatocytes. This multifunctional protein was recently identified as a factor associated with pregnancy complications such as spontaneous preterm delivery, preeclampsia, and gestational diabetes [2]. Aside from its classical function in vitamin D transport, VDBP promotes actin clearance during tissue remodeling following both physiological and pathological cell death [7]. VDBP also engages with many immune cells [8–10] and is a precursor of macrophage activating factor (VDBP-MAF) [11, 12]. Thus, VDBP is capable of modifying inflammation and protecting against vascular dysfunction. Since inflammation and vascular dysfunction are pathological features of the placenta in fetal growth restriction (FGR), we hypothesized that placental VDBP expression levels are altered in FGR. To address this hypothesis, both VDBP and vitamin D 25(OH)D stores were measured in placentae from FGR-affected and gestation age-matched uncomplicated pregnancies.

## 2. Methods

Informed consent was obtained from all participants following institutional human research ethics approval. FGR inclusion criteria were reduced birthweight [<10th centile on contemporary growth charts [13]] and at least one pathological marker, growth asymmetry (head-to-abdominal circumference ratio ≥ 1.2), reduced amniotic fluid index (≤7 cm), abnormal umbilical artery Doppler (elevated systolic/diastolic ratio > 95th centile or absent end-diastolic flow), and/or impaired growth trajectory (>30% growth centile fall during the third trimester). Nonidiopathic FGR was defined by the presence of FGR-associated comorbidities (including preeclampsia, chronic hypertension, gestational diabetes, smoking, and alcohol abuse), and idiopathic FGR was defined by the absence of these confounding features. A total of 35 placentae were investigated, 17 from normal healthy pregnancies and 18 derived from pregnancies complicated by FGR. The FGR group consisted of 9 idiopathic and 9 nonidiopathic pregnancies.

### 2.1. VDBP and 25(OH)D Enzyme-Linked Immunosorbent Assays (ELISA)

Protein was extracted from gestation-matched third-trimester placentae derived from uncomplicated pregnancies and pregnancies complicated by FGR and the protein concentration of each placental sample was assayed as described previously [14]. Briefly, 500 mg of placenta was homogenized in a 50 mM glycine buffer (Bio-Rad, Hercules, CA, USA) with 0.5% Triton X-100 (BDH, Victoria, Australia), 1 mM AEBSF (ICN, New South Wales, Australia), and 5 mM EDTA and centrifuged (10 min, 3500 rpm, 4°C). The supernatant was aliquoted in small volumes (50 *μ*l) to minimize repeated freeze/thaw cycles of samples and stored at −40°C for up to 2 years. Placental VDBP content was determined using the DuoSet® ELISA kit (R&D Systems, Minneapolis, MN) and normalized to total protein concentration. Vitamin D measurements were performed using the 25(OH) vitamin D ELISA kit (IBL International, Hamburg, Germany) and normalized to total protein concentration.

### 2.2. Statistical Analysis

All data are expressed as mean ± SEM. Categorical data regarding patient characteristics were analyzed using a 2 × 2 contingency table with Fisher's Exact Test or 3 × 2 contingency table with Chi-Squared Test. Parametric data from patient characteristics and VDBP measurements were analyzed using unpaired Student's *t*-test, with Welch's correction to account for unmeasured biological variability. Data was assumed to be normally distributed according to the Kolmogorov-Smirnov normality test (*p* > 0.05; data not shown), with the exception of the idiopathic FGR group (*p* < 0.05). For analyses involving the latter group, the Kruskal-Wallis test with Dunn's multiple comparisons was applied. Associations between parametric data (placental VDBP versus vitamin D content) were determined using Pearson's correlation on GraphPad Prism 7 software, with statistical significance deemed at *p* < 0.05.

## 3. Results and Discussion

**Figure 1:**
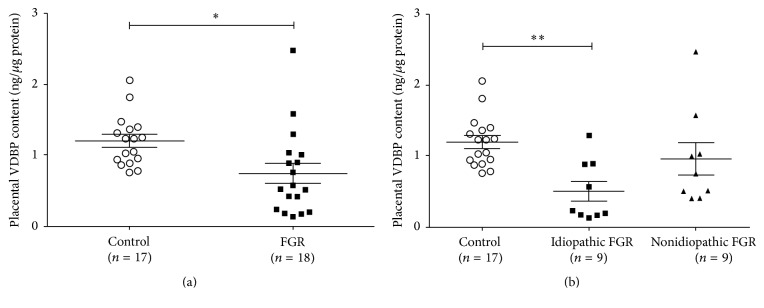
(a) Quantification of VDBP protein content in third-trimester placenta samples from control and FGR pregnancies. ^*∗*^*p* < 0.05, unpaired Student's *t*-test with Welch's correction. (b) The same data shown in panel (a) but with data from FGR pregnancies separated into idiopathic and nonidiopathic FGR ^*∗∗*^*p* < 0.01, Kruskal-Wallis test with Dunn's multiple comparisons. Data presented as mean ± SE.

Clinical inclusion criteria for FGR are summarized in Table 1, whilst Table 2 summarizes the demographic and obstetric data for all participants. With the exception of fetal birthweight and placental weight, there were no significant differences between control participants and those with an FGR-affected pregnancy. This study is the first to demonstrate significantly reduced placental VDBP concentration in pregnancies complicated by FGR compared to uncomplicated pregnancies (*p* < 0.05; Figure 1(a)). Furthermore, these data suggest that reduced VDBP is most pronounced in the idiopathic FGR subgroup (*p* < 0.01; Figure 1(b)). As such, VDBP may be a factor in unexplained placental insufficiency although further functional studies are needed to explore causality in this context.

**Figure 2:**
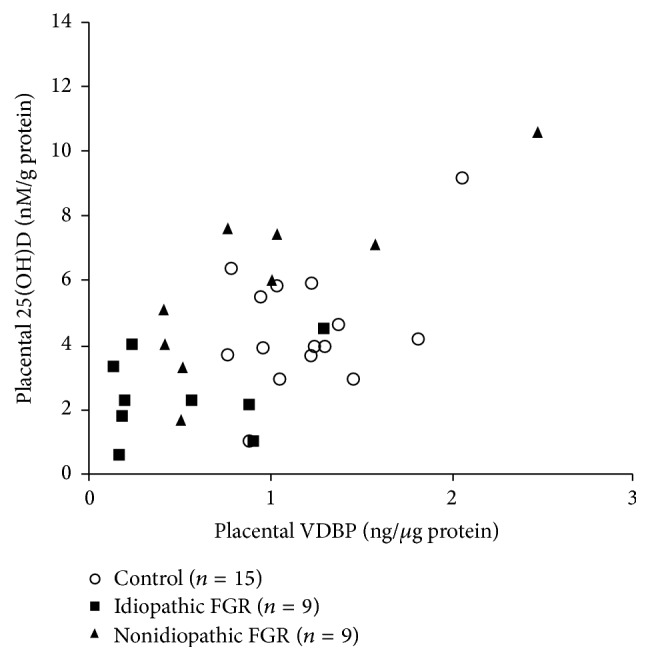
Relative VDBP and 25(OH) vitamin D concentrations do not significantly correlate, as measured in third-trimester placentae from control and FGR pregnancies, *n* = 35, *p* = 0.295, *r* = 0.182, Pearson's correlation.* Note*. two extreme outliers from the control 25(OH) vitamin D group have been excluded from the graph.

Correlation analysis indicated that placental VDBP concentrations were independent of 25(OH) vitamin D content (*n* = 35, *p* = 0.295, Figure 2). From this, we hypothesize that placental actions of VDBP are unrelated to placental vitamin D function. However, this analysis was limited to the vitamin D storage form, 25(OH)D, and not the active form 1,25(OH)_2_D. Whilst 1,25(OH)_2_D is less stable and difficult to reliably quantify, it correlates with specific VDBP isoforms in serum and other tissues [15]. Furthermore, 25(OH) vitamin D measurements were performed on samples obtained after delivery, well after the vitamin D expression peak and establishment of placental function [16]. Indeed, first-trimester tissue (e.g., chorionic villus samples) may provide insight into the role of VDBP in the etiology of FGR.

VDBP may affect placental function through nutrient transport [6], placental perfusion [17], chronic inflammation, or any combination of these. VDBP has the capacity to modulate placental inflammatory processes, including removal of apoptotic debris and villus remodeling [18]. Given the well-recognised role of inflammation in the course of placental maldevelopment and malfunction, it is plausible that low VDBP may have preceded these changes. Future studies should focus on the other member of the actin-scavenging system (gelsolin) and on defining a placental VDBP and gelsolin profile throughout gestation.

Aside from downstream functional effects, it is important to consider determinants of placental VDBP content. Factors affecting VDBP uptake into the placenta deserve research attention. Future studies should incorporate patient-matched serum samples, to determine whether VDBP changes are unique to the placenta. In addition, VDBP has three main alleles (and over 120 rare genetic variants) [19] which affect turnover rates and serum VDBP concentration [20]. These also influence vitamin D- [4] but not actin-binding actions [21]. Functional data in pregnancy are limited, though specific VDBP variants have been linked to gestational diabetes [22] and preterm labor [23, 24]. Analysis of VDBP functional variants in the setting of FGR could therefore be highly informative. Indeed, there may be a need to evaluate VDBP measurements according to function rather than quantity alone.

## 4. Conclusions

Placental expression of vitamin D-binding protein from pregnancies complicated by fetal growth restriction was assessed. VDBP was significantly reduced and strongly associated with idiopathic fetal growth restriction. There was no association between placental VDBP and 25(OH) vitamin D stores. VDBP may be a factor in unexplained placental dysfunction associated with idiopathic fetal growth restriction and may potentially serve as a surrogate marker of this disease.

## Figures and Tables

**Table 1:** Clinical inclusion criteria for FGR.

Patient characteristics	FGR-affected^*∗*^ (*n* = 18)
Fetal birthweight	
<10th centile	18 (100%)
<3rd centile	11 (61%)

Fetal growth pattern (HC : AC ratio)	
Asymmetrical (≥1.2)	10 (56%)
Symmetrical (<1.2)	8 (44%)

Amniotic fluid index	
Abnormal (≤7)	9 (50%)
Normal (>7)	9 (50%)

Umbilical artery Doppler	
Abnormal (S : D ratio > 95th percentile or absent EDF)	8 (44%)
Normal (S : D ratio < 95th percentile)	10 (56%)

Fetal growth trajectory	
Significantly impaired (≥30% drop during the third trimester)	2 (11%)
Adequate (<30% deviation)	16 (89%)

Maternal factors	
Gestational diabetes	1 (6%)
Preeclampsia	1 (6%)
Chronic hypertension	1 (6%)
Smoking	5 (28%)
Alcohol abuse	1 (6%)

HC : AC: head circumference : abdominal circumference ratio.

S : D: systolic : diastolic ratio.

EDF: end-diastolic flow.

^*∗*^Patients may fulfil more than one category.

**Table 2:** Patient demographic and obstetric characteristics.

Patient characteristics^a^	Control (*n* = 17)	FGR-affected (*n* = 18)	*p* value
Maternal age (yrs)	32.12 (±1.33)	30.44 (±1.65)	0.43^b^

Parity			
Primiparous	6	11	0.18^c^
Multiparous	11	7	

Gestational age (wks)	35.47 (±1.03)	36.56 (±0.64)	0.38^b^

Birthweight centile (%)	65.85 (±5.90)	3.06 (±0.64)	<0.001^b^

Placental weight^d^	583.36 (±32.37)	399.88 (±25.19)	<0.001^b^

Infant sex			
Female	11	8	0.31^c^
Male	6	10	

Mode of delivery			
Vaginal	4	5	0.79^c^
Caesarean (in labour)	1	2	
Caesarean (not in labour)	12	11	

^a^Data presented as the mean (±SEM).

^b^Student's *t*-test with Welch's correction was used for parametric data.

^c^2 × 2 contingency table with Fisher's Exact Test or a 3 × 2 contingency table with Chi-Squared Test was used (where appropriate) for categorical data.

^d^Placental weights for *n* = 3 controls and *n* = 2 FGR-affected pregnancies were not recorded.

## Abbreviations

FGR: Fetal growth restriction
VDBP: Vitamin D-binding protein.
